# Pneumococcal Conjugated Vaccine Reduces the High Mortality for Community-Acquired Pneumonia in the Elderly: an Italian Regional Experience

**DOI:** 10.1371/journal.pone.0166637

**Published:** 2016-11-15

**Authors:** Vincenzo Baldo, Silvia Cocchio, Tolinda Gallo, Patrizia Furlan, Pierantonio Romor, Chiara Bertoncello, Alessandra Buja, Tatjana Baldovin

**Affiliations:** 1 Department of Cardiac, Thoracic, and Vascular Sciences, Hygiene and Public Health Unit, University of Padua, Padua, Italy; 2 EuroHealth Net, Friuli Venezia Giulia Region Health Directorate, Udine, Italy; Universidade Federal de Sao Paulo, BRAZIL

## Abstract

**Background:**

Community-acquired pneumonia (CAP) is an important cause of illness and death worldwide, particularly among the elderly. Previous studies on the factors associated with mortality in patients hospitalized for CAP revealed a direct association between the type of microorganism involved, the characteristics of the patient and mortality. Vaccination status against pneumococcal disease was not considered. We conducted a retrospective analysis on the mortality rates after a first hospitalization for CAP in north-east Italy with a view to examining especially the role of anti-pneumococcal vaccination as a factor associated with pneumonia-related mortality at one year.

**Method:**

Between 2012–2013, patients aged 65+ hospitalized with a primary diagnosis of CAP, identified based on International Classification of Diseases, Ninth Revision, Clinical Modification codes 481–486, were enrolled in the study only once. Patients were divided into three groups by pneumococcal vaccination status: 1) 13-valent pneumococcal conjugate vaccine (PCV13) prior to their hospitalization; 2) 23-valent pneumococcal polysaccharide vaccine (PPV23) within 5 years before hospitalization and 3) unvaccinated or PPV23 more than 5 years prior to admission. Gender, age, length of hospital stay and influenza vaccination were considered. Comorbidities were ascertained by means of a properly coded diagnosis. Every patient was followed up for 1 year and the outcome investigated was mortality for any cause and for pneumonia.

**Results:**

A total of 4,030 patient were included in the study; mean age at the time of admission to hospital was 84.3±7.7; 50.9% were female. 74.2% of subjects had at least one comorbidity; 73.7% has been vaccinated against influenza. Regard to pneumococcal vaccine, 80.4% of patients were not vaccinated, 14.5% vaccinated with PPV23 and 5.1% with PCV13. The 1-year survival rates after hospitalization for pneumonia were 83.6%, 85.9% and 89.3% in the unvaccinated, PPV23 and PCV13 groups, respectively. Regression analysis indicated that the risk of death due to pneumonia increased significantly with age (adjusted OR: 1.073; 1.061–1.085), shorter hospital stay (adjusted OR: 0.981; 0.971–0.990), and male gender (adjusted OR: 1.372; 1.165–1.616). The model also confirmed the pneumococcal 13-valent conjugated vaccine as an independent protective factor for mortality-related pneumonia (adjusted OR: 0.599; 0.390–0.921).

**Conclusion:**

The main finding of our observational cohort study is a high mortality rate among elderly patients admitted to hospital for pneumonia. The present study suggests a protective role for PCV13 vaccination.

## Introduction

Community-acquired pneumonia (CAP) is an important cause of illness and death worldwide, particularly among the elderly [1]. *Streptococcus pneumoniae* (SP) is the most common bacterial cause of CAP among adults in industrialized countries, and pneumococcal infections are considered a major global public health issue for all age groups. The pathogens reportedly identified as being responsible for pneumonia may vary, however, depending on the equipment and/or expertise available at in-hospital laboratories, the study designs used in published reports, the seasons of the year, and the regions where studies are conducted [2].

The epidemiological picture of CAP-related hospitalization trends varies, with some countries recording a drop in the rates due to herd effects among elderly, while others report rising numbers of hospital admissions for CAP, possibly as a result of the rising proportions of elderly people in the general populations, and their higher prevalence of concomitant clinical conditions (such as chronic obstructive pulmonary disease or diabetes) [3–7]. Whatever a region’s CAP-related hospitalization rate, about 70% of cases involve patients aged 65 years and older [8, 9].

CAP is associated with mortality rates that reflect the site of patient care: while outpatients have a <5% risk of dying, hospitalized cases have a mortality rate of 12%, and the chances of death for patients admitted to the intensive care unit exceeds 30% [10]. It is very important to assess the severity of pneumonia cases accurately to ensure their appropriate management, given its impact on the related mortality risk and on the cost of care for these patients.

Previous studies on the factors associated with mortality in patients hospitalized for CAP revealed a direct association between the type of microorganism involved, the characteristics of the patient (age, sex and comorbidities) and mortality [8]. Vaccination status against pneumococcal disease was not considered, however, due to a shortage of data because PCV13 vaccination in the elderly was introduced only recently.

We conducted a retrospective analysis of the mortality rates after a first hospitalization for CAP in north-east Italy with a view to examining especially the role of anti-pneumococcal vaccination as a factor associated with pneumonia-related mortality at one year.

## Material and Methods

From 1 January 2012 to 31 December 2013, a retrospective observational cohort study was conducted on data routinely collected by the health services in Friuli Venezia Giulia (FVG), a region of north-eastern Italy. At the time of the study, FVG had a population of about 1.2 million with an average age of 46.2 years, an old-age index of 191.8%, and a mortality rate of 11.2 per 1,000 population. The region had 4,226 hospital beds, about 3.5 per 1,000 population. The overall hospitalization rate was 106.1 per 1,000 population, for a hospital bed occupation rate of 76.5% and a mean hospital stay of 7.2 days.

### Setting

The FVG Regional Authority operates an automated, centralized system for recording and pooling data on health care funded by the Italian National Health Service, and patients are identified in the database by means of unique anonymous personal codes. The following health data were used for this study: hospital discharge records (HDRs, coded according to the *International Classification of Diseases*, *Ninth Revision*, *Clinical Modification* [ICD-9-CM]) of all public and accredited private hospitals; mortality records (MR, coded according to the ICD-9-CM); health-related tax exemptions (HTEs; coded using an Italian national coding system); drug prescriptions (DPs; based on the Anatomical Therapeutic Chemical classification system [ATC]); and the FVG regional cancer and vaccination registries (VR).

An active program of immunization for the elderly with 13-valent pneumococcal conjugate vaccine (PCV13) was first introduced in FVG in 2012, when PCV13 was adopted instead of the 23-valent pneumococcal polysaccharide vaccine (PPV23). Pneumococcal vaccination was offered in co-administration with influenza vaccine.

### Patients

Patients aged 65 years older who were hospitalized with a primary diagnosis of pneumonia, based on ICD-9-CM codes 481–486 (CAP-related hospitalizations) were enrolled in the study only once, on their first hospitalization (i.e. they had not been admitted to hospital for pneumonia during the previous year).

Pneumonia was diagnosed on the grounds of instrumental, clinical and microbiological findings, and a specific ICD code was assigned as follows: 481 for *Streptococcus pneumoniae*, 482 for other bacterial pneumonia (*Klebsiella pneumoniae*, *Pseudomonas*, *Haemophilus influenza*, *Streptococcus spp*, *Staphylococcus and other specified bacteria*), 483 for pneumonia due to other specified organism (*Mycoplasma pneumoniae* and *Chlamydia*), 484 for pneumonia in infectious diseases classified elsewhere, and 485–486 for pneumonia without a causative organism identified.

Patients were divided into three groups by pneumococcal vaccination status. Group 1 included patients vaccinated with PCV13 prior to their hospitalization; Group 2 concerned those vaccinated with PPV23 within 5 years before hospitalization (based on data on the duration of vaccine-induced protection [11]); and Group 3 included patients who had never been vaccinated, and those vaccinated with PPV23 more than 5 years prior to admission. Influenza vaccination was considered if it had been performed no more than one year before the patient’s hospitalization.

### Comorbidity

For all subjects, comorbidities were identified by means of their HDRs, HTEs, and DPs recorded between 2000 and 2011. Patients with asthma were identified from HDRs containing a diagnosis (primary or secondary) of asthma (ICD-9-CM code: 493*), or from prescriptions for anti-asthma drugs (ATC code: R03A, R03CC02, R03CC04, R03CK, R03DC01, R03DC03), or HTEs for asthma (code: 007.493). Cases of chronic obstructive pulmonary disease (COPD) were identified from HDRs with ICD-9-CM codes 490*, 491*, 492*, 494* and 496*. Cases of diabetes mellitus were identified from HDRs with ICD-9-CM codes 250* or at least two DPs for antidiabetic drugs (ATC code: A10A* and A10B*) at two different times, or HTEs (code: 013.250). Cases of cancer were identified from the FVG cancer registry.

### Outcome

Deaths during the follow-up were ascertained from the records in the FVG Cause of Death Register. The cause of death of residents of the region is reported on death certificates by the medical examiners, then ICD coded and validated by personnel at the local health agencies. The coverage of this register is 100%. Mortality was recorded during a 1-year follow-up after a pneumonia-related hospital stay, and was classified as: in-hospital; at 30 days after discharge; and at 1 year after discharge. All deaths were included in one of five groups based on the main cause of death depending on the organ system involved, as described by Bruns et al. [12]: (i) pneumonia (ICD: 481–486); (ii) COPD and allied conditions (ICD: 490–496); (iii) vascular disease (ICD: 390–459); (iv) malignancy (ICD: 140–239); and (v) others (all other ICD).

### Statistical analysis

A descriptive analysis was conducted on patients’ demographics and clinical data. Data were presented as percentages for categorical variables and compared using the chi-square test or Fisher’s exact test, or as means ± standard deviations (SD) for continuous variables, which were compared using Student’s t-test for unpaired data, performing a priori tests for equality of variances. The mortality rate was calculated by group, with the corresponding 95% CIs. Patient survival after hospital discharge was assessed with a Kaplan-Meier survival analysis. Survival curves were compared using log-rank and Wilcoxon’s test. Mortality risk factors were analyzed with Cox’s regression model.

The mortality observed in our cohort was compared with the expected total mortality in the Friuli Venezia Giulia region’s population aged 65 years over hospitalized during the study period for any reason (with no other hospital admissions in the previous year), stratified by age and gender. This enabled us to estimate standardized mortality ratios (SMRs) with 95% Confidence Interval (95% CI).

A P-value of <0.05 was accepted as statistically significant. The analyses were performed using the Statistical Package for the Social Sciences (SPSS 22.0; SPSS Inc., Chicago, IL, USA).

### Ethics statement

Data were treated with full confidentiality in accordance with Italian legislation. Before the database was made available to the authors, patient identifiers were replaced with anonymous codes that make it impossible to identify the individuals concerned. It was unnecessary to obtain patients’ informed consent, given the anonymous nature of the data and its mandatory recording (anonymized data may be analyzed and used in aggregate form for scientific studies without further authorization) [13]. This study complies with the Declaration of Helsinki and the study protocol was approval by the ethical committee of the Padua Provincial Authority.

## Results

During the study period, 79,294 all-cause hospitalizations were extrapolated from the regional HDRs. Among all these hospital admissions, 4,766 (6.0%) were CAP-related, but 736 of these cases were excluded from the study due to patients having being vaccinated with a pneumococcal vaccine after their discharge from hospital. Among the remaining 4,030 patients included in our study, a specific pneumonia diagnosis was mentioned for 6.6% of admissions (*Streptococcus pneumoniae* in 2.7%, other bacterial pneumonia in 3.9%), while the etiology was not specified for 93.4%. There was a slight majority of females (50.9%). The mean age of the sample at the time of admission to hospital was 84.3±7.7, and 70.2% of the patients were at least 80 years old. In the cohort as a whole, 73.7% had been vaccinated against influenza; 74.2% patients had at least one comorbidity, and males were significantly more affected by all the comorbidities analyzed (p<0.05). The mean hospital stay was 12.7±11.1 days.

**Fig 1:**
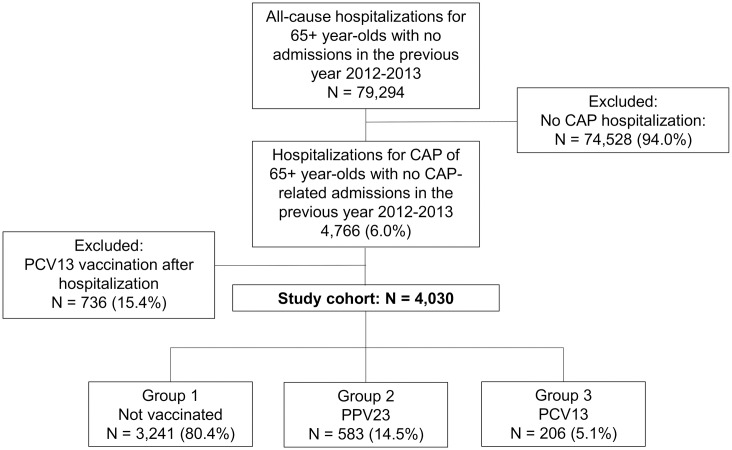
Patient selection.

The flow-chart in Fig 1 summarizes the patient and study group selection process.

During the one-year follow-up, the deaths observed among patients aged 65 or older who were hospitalized for CAP amounted to 2,291, as opposed to an expected 1,057.3 deaths based on the regional rate, which results in a SMR for our cohort of 2.17 (95% CI: 2.08–2.26). The SMR was 2.20 (95% CI: 2.07–2.32) for males, and 2.07 (95% CI: 1.95–2.19) for females. The SMRs by time of death are shown in Table 1.

**Table 1:** Standardized mortality rates (SMR) by gender and time of death.

Time of death	Male		Female		Total	
	SMR	95% CI	SMR	95% CI	SMR	95% CI
In-hospital mortality	3.01	5.22–6.24	2.66	2.42–2.89	2.86	2.68–3.04
30-day mortality	2.24	1.92–2.59	2.19	1.89–2.54	2.25	2.02–2.49
1-year mortality	1.73	1.58–1.88	1.65	1.51–1.81	1.73	1.62–1.83
Cumulative mortality	2.20	2.07–2.32	2.07	1.95–2.19	2.17	2.08–2.26

Table 2 shows the distribution of the main causes of death by time of death. Within 30 days after discharge, respiratory disease was the leading cause of death, with 540 patients dying of pneumonia, and 48 due to COPD (40.9% pneumonia alone and 44.5% pneumonia plus COPD, respectively). At 1 year after discharge, on the other hand, vascular diseases had become the leading cause of death (36.0%). Of the 639 of all deaths within 1 year due to vascular diseases, 48.6% were due to heart disease, 30.8% to cerebrovascular disease, 13.1% to hypertensive disease, and 7.4% to other diseases of the circulatory system.

**Table 2:** Distribution of 2,291 patients who died by time and cause of death.

Cause of death	In-hospital		At 30 days after discharge		At 1 year after discharge		Cumulative mortality	
	n	(%)	n	(%)	n	(%)	n	%
Pneumonia	480	(49.4)	60	(17.2)	95	(9.8)	635	(27.7)
COPD and allied conditions	37	(3.8)	11	(3.2)	42	(4.3)	90	(3.9)
Vascular disease	171	(17.6)	118	(33.9)	350	(36.0)	639	(27.9)
Malignancy	47	(4.8)	56	(16.1)	176	(18.1)	279	(12.2)
Other	237	(24.4)	103	(29.6)	308	(31.7)	648	(28.3)
All causes	972		348		971		2,291	

As concerns pneumococcal vaccination status, 80.4% of patients were not vaccinated, 14.5% were vaccinated with PPV23, and 5.1% with PCV13 (Fig 1). The subjects administered PPV23 vaccine were of significantly younger age than the other two groups (83.3±8.1 years old in the PPV23 group versus 84.7±7.4 in the PCV13 group, and 84.4±7.7 for the unvaccinated subjects; p<0.05). The frequency of comorbidities was significantly higher among subjects vaccinated with PCV13 (p<0.05). The rate of vaccination against influenza virus was significantly higher among subjects also vaccinated against pneumococcal disease (p<0.05). The cumulative all-cause mortality rate was lower among patients vaccinated with PCV13 (102 deaths, 49.5%), than among the unvaccinated patients (1,857 deaths, 57.3%; p<0.05) or the group vaccinated with PPV23 (332 deaths, 56.9%; p<0.05). Table 3 shows the demographics and clinical features of patients grouped by pneumococcal vaccination status.

**Table 3:** Characteristics of the study population by pneumococcal vaccination status.

Variables	Pneumococcal vaccination status					
	Not vaccinated		PPV23		PCV13	
	n	(%)	n	(%)	n	(%)
Gender [n(%)]						
Males	1,577	(48.7)	293	(50.3)	107	(51.9)
Females	1,664	(51.3)	290	(49.7)	99	(48.1)
Age groups [n(%)]						
65–69	112	(3.5)	29	(5.0)	3	(1.5)
70–74	339	(10.5)	87	(14.9)	21	(10.2)
75–79	479	(14.8)	96	(16.5)	34	(16.5)
80–84	643	(19.8)	91	(15.6)	41	(19.9)
85+	1,668	(51.5)	280	(48.0)	107	(51.9)
At least one comorbidity [n(%)]	2,380	(73.4)	440	(75.5)	169	(82.0)
Asthma	1,253	(38.7)	235	(40.3)	94	(45.6)
COPD	583	(18.0)	116	(19.9)	48	(23.3)
Chronic heart diseases	1,237	(38.2)	233	(40.0)	78	(37.9)
Diabetes	792	(24.4)	162	(27.8)	56	(27.2)
Malignant neoplasms	518	(16.0)	99	(17.0)	30	(14.6)
Influenza vaccination [n(%)]	2,251	(69.5)	526	(90.2)	204	(99.0)
All-cause mortality						
in-hospital	803	(24.8)	133	(22.8)	36	(17.5)
at 30 days	282	(8.7)	52	(8.9)	14	(6.8)
at 1 year	772	(23.8)	147	(25.2)	52	(25.2)
cumulative	1,857	(57.3)	332	(56.9)	102	(49.5)

**Fig 2:**
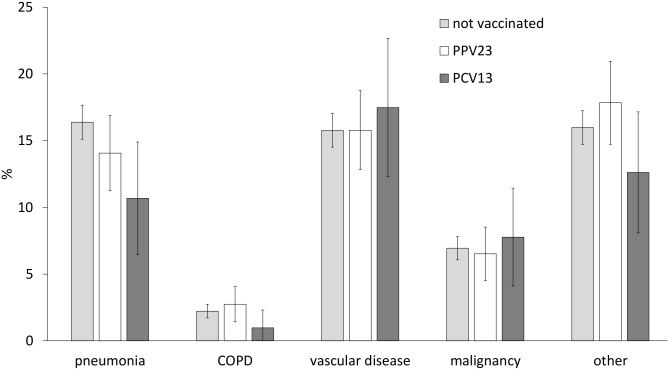
Mortality rate (%) by cause of death and pneumococcal vaccination status.

Fig 2 shows the distribution of the causes of death by pneumococcal vaccination status. Among the deaths due specifically to pneumonia, a mortality rate of 10.7% (95% CI: 6.5%-14.9%) was calculated in the PCV13 group, which was lower than in the unvaccinated or PPV23 groups, with 16.4% (95% CI: 15.1%-17.7%) and 14.1% (95% CI: 11.2%-16.9%), respectively.

**Fig 3:**
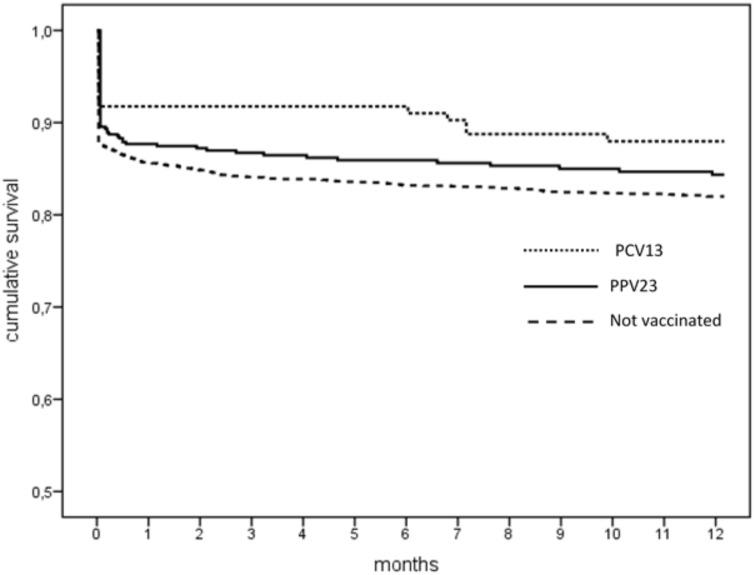
One-year survival after pneumonia by patients’ vaccination status.

Specifically as concerns pneumonia, the survival rates at 1-year after hospitalization were 83.6%, 85.9% and 89.3% in the unvaccinated, PPV23 and PCV13 groups, respectively. The mean survival for this cause of death was 10.2±0.1 months in the unvaccinated group, 10.5±0.2 months in the PPV23 group, and 10.9± 0.2 months in the PCV13 group (Fig 3).

Regression analysis indicated that the risk of death due to pneumonia increased significantly with age (adjusted OR: 1.073; 95% CI: 1.061–1.085), shorter hospital stays (adjusted OR: 0.981; 95% CI: 0.971–0.990), and male gender (adjusted OR: 1.372; 95% CI: 1.165–1.616). The model also confirmed the pneumococcal 13-valent conjugated vaccine as an independent protective factor against pneumonia mortality (adjusted OR: 0.599; 95% CI: 0.390–0.921).

## Discussion

The main finding of our observational cohort study is that hospital admission for CAP in the elderly was associated with a higher mortality rate than hospitalization for other illnesses. We found the pneumonia-related mortality 2.17-fold higher than was to be expected judging from the overall hospitalization data, adjusted for age and gender. The difference was greater for in-hospital and 30-day mortality, then dropped to a 1.73-fold increase in one-year mortality. The consistently high all-cause mortality rate for patients with CAP observed in our population reflects the findings of other studies, and may represent an accurate estimate of the mortality risk associated with an episode of CAP among people over 65 [14].

Several studies have reported one-year mortality rates for subjects hospitalized for CAP in the range of 7% to 41%, depending on the population considered [2, 15–21]. Our population was very elderly and 75% of these patients had chronic conditions such as asthma and chronic heart disease. Our 30-day mortality rate could be due to the fact that only the more severe cases of pneumonia are hospitalized, whereas most patients developing pneumonia in our geographical area are not admitted to hospital. In fact, our region has lower hospitalization rates for pneumonia than other Italian regions, but a higher case fatality rate [22].

The cause of death differed, depending on the time of its occurrence. Pneumonia was the main cause of early death, confirming that hospital admission was reserved for the most severe cases. Later causes of death were attributed mainly to chronic conditions such as vascular disease, and malignancies, not to recurrent pneumonia, a finding consistent with previous studies [13–14, 23–24]. As in other reports, our findings further support the impression that cardiovascular events may play an important part in the long-term outcome of hospital survivors of CAP [25–27].

Our sample revealed a better one-year survival rate among subjects who had been immunized with conjugate vaccine against pneumococcal disease prior to their admission to hospital. Their lower mortality over the study period is attributable mainly to a lower death rate related to lung disease, especially when pneumonia was considered alone. The one-year survival rate after hospitalization for pneumonia was significantly higher (about 7%) for the PCV13-immunized patients than for the other two groups. Our analysis revealed a protective role of pneumococcal vaccination to survival of subjects. As for the other factors investigated, advanced age, male sex, and a shorter hospital stay were also associated with a lower one-year survival rate after hospitalization for pneumonia.

There is currently a shortage of information on the role of PCV13 vaccination for adults in protecting against mortality due to pneumococcal disease [28]. Trials conducted on pneumococcal polysaccharide vaccines have failed to provide evidence to support the routine use of PPVs to prevent all-cause pneumonia or the related mortality, though some studies indicate that PPVs confer protection against invasive pneumococcal disease (IPD) [29–30]. The present study is one of the few in which outcome was assessed in an open population in relation to patients’ vaccination status. Our findings warrant further investigation in studies with a longer follow-up and larger samples of subjects.

The results of the CAPITA study indicated that vaccination is effective in reducing CAP and IPD in individuals over 65 years old with no chronic comorbidities [31]. Our findings suggest instead that vaccination is effective in preventing fatalities among patients hospitalized for an episode of severe CAP. The two studies were conducted in very different settings, since our sample was generally frail, and our follow-up began with the episode of CAP and should be further investigated to see how elderly patients with chronic diseases may benefit in survival terms.

Our study has some limitations, the first of which concerns the small number of patients in the PCV13-vaccinated group: this could be partly explained by the protective effect of immunization against CAP and IPD (as seen in the CAPITA trial), which would consequently reduce the likelihood of hospitalization for PCV13-vaccinated patients with CAP because their disease would be less severe and they would be managed as outpatients. Further studies could therefore focus on the role of vaccination in reducing CAP-related hospitalizations. A second possible limitation lies in that HDRs provide no microbiological data, though, according to the literature, an estimated 27.3% of cases of CAP are attributable to *Streptococcus pneumoniae* [32], and consequently potentially preventable by means of vaccination.

The strengths of our work lie in having designed our retrospective cohort study to investigate whether pneumonia is an event that increases short- or long-term mortality in a well-defined patient population by means of data routinely collected by the public health service, and following patients up for up to 1 year after their discharge from hospital. The analysis of routinely-available records can prove an efficient method for monitoring trends in the state of health of particular populations.

## Conclusions

The results of the present study confirm a higher mortality rate among elderly people hospitalized for pneumonia and suggest a protective role for PCV13 vaccination. Long-term studies are needed, however, to confirm the benefit of PCV13 in reducing pneumonia-related mortality in the elderly. Meanwhile, efforts should be made to improve vaccination programs for the elderly, given the higher frequency of chronic diseases in this population.
